# CHIP is a novel tumor suppressor in pancreatic cancer and inhibits tumor growth through targeting EGFR

**DOI:** 10.18632/oncotarget.1890

**Published:** 2014-04-08

**Authors:** Tianxiao Wang, Jingxuan Yang, Jianwei Xu, Jian Li, Zhe Cao, Li Zhou, Lei You, Hong Shu, Zhaohui Lu, Huihua Li, Min Li, Taiping Zhang, Yupei Zhao

**Affiliations:** ^1^ Department of General Surgery, Peking Union Medical College Hospital, Chinese Academy of Medical Sciences and Peking Union Medical College, Beijing, China; ^2^ The Vivian L. Smith Department of Neurosurgery, the University of Texas Medical School at Houston, Houston, Texas, USA; ^3^ Department of Pathology and Pathophysiology, Peking Union Medical College Hospital, Chinese Academy of Medical Sciences and Peking Union Medical College, Beijing, China; ^4^ Department of Pathology and Pathophysiology, School of Basic Medical Sciences, Capital Medical University, Beijing, China

**Keywords:** CHIP, EGFR, pancreatic cancer, ubiquitination

## Abstract

Carboxyl terminus of heat shock protein 70-interacting protein (CHIP) is an E3 ubiquitin ligase that is involved in protein quality control and mediates several tumor-related proteins in many cancers, but the function of CHIP in pancreatic cancer is not known. Here we show that CHIP interacts and ubiquitinates epidermal growth factor receptor (EGFR) for proteasome-mediated degradation in pancreatic cancer cells, thereby inhibiting the activation of EGFR downstream pathways. CHIP suppressed cell proliferation, anchor-independent growth, invasion and migration, as well as enhanced apoptosis induced by erlotinib *in vitro* and *in vivo*. The expression of CHIP was decreased in pancreatic cancer tissues or sera. Low CHIP expression in tumor tissues was correlated with tumor differentiation and shorter overall survival. These observations indicate that CHIP serves as a novel tumor suppressor by down-regulating EGFR pathway in pancreatic cancer cells, decreased expression of CHIP was associated with poor prognosis in pancreatic cancer.

## INTRODUCTION

Pancreatic cancer (PC) is the fourth leading cause of cancer-related deaths in the United States [1] with an incidence rate that is nearly equal to its mortality rate, which demonstrates the aggressiveness and lethal nature of this disease. The overall 5-year survival rate is found to be <6%[2]. Locally advanced tumors with metastatic disease are often considered to be advanced pancreatic cancer with poor prognosis. Given the low overall response rates to traditional chemotherapy, novel therapeutic targets are urgently needed for this malignant disease.

EGFR is a transmembrane glycoprotein that is conserved and overexpressed in pancreatic cancer[3, 4]. It is a member of the ErbB family of receptors and has tyrosine kinase activity. The phosphorylation of EGFR initiates downstream signaling cascade, such as MAPK, PI3K/Akt and Src pathways, which have been implicated in carcinogenesis by affecting cell proliferation, survival, invasion and metastasis[5]. EGFR over-expression is thought to confer a poor survival, correlating with a more advanced stage and the presence of metastases in pancreatic cancer. Therefore, inhibition of the EGFR signaling pathway is an attractive therapeutic target. Erlotinib is a small molecule tyrosine kinase inhibitor (TKI) that selectively inhibits EGFR activation. A phase III study demonstrated a significant survival benefit associated with this targeted agent combined with gemcitabine in advanced pancreatic cancer [6]. However, previous reports have established that patients rapidly developed resistance, which was most likely caused by a shorter EGFR intron 1 CA repeat length [7], the mutation of KRAS[8], and the amplification of c-Met[9] in pancreatic cancer or other tumors.

CHIP is a U-box dependent E3 ubiquitin ligase that functions as a chaperone for protein quality control and as a ubiquitin ligase that degrades its substrates with the help of proteasome machinery. The structure of CHIP is composed of a tetratricopeptide repeat domain (TPR) that links to the chaperones Hsp70/Hsp90, a charged domain, and a U-box domain that is essential for E3 ubiquitin ligase activity. Increased evidence showed that CHIP not only modulates misfolded proteins but also regulates pathophysiological processes. CHIP is associated with many tumor-related proteins, such as ErbB2 [10], c-Met[11], SRC-3[12], NF-κB[13], AKT[14], PTEN[15] and p53[16]. The up-regulation of CHIP could inhibit tumor growth and metastasis, and its levels were negatively correlated with the malignancy of human breast or gastric tumors. However, the exact mechanisms of CHIP in pancreatic cancer have not been elucidated to date. In the present study, we identified that EGFR, a Hsp90 client, is regulated by CHIP through ubiquitination in pancreatic cancer cells. We also investigated the functions of CHIP in pancreatic tumor progression and the significance of CHIP levels in sera or tissues of pancreatic cancer patients.

## RESULTS

### CHIP regulates EGFR levels through the Ub-Proteasome pathway in pancreatic cancer cells.

**Figure 1:**
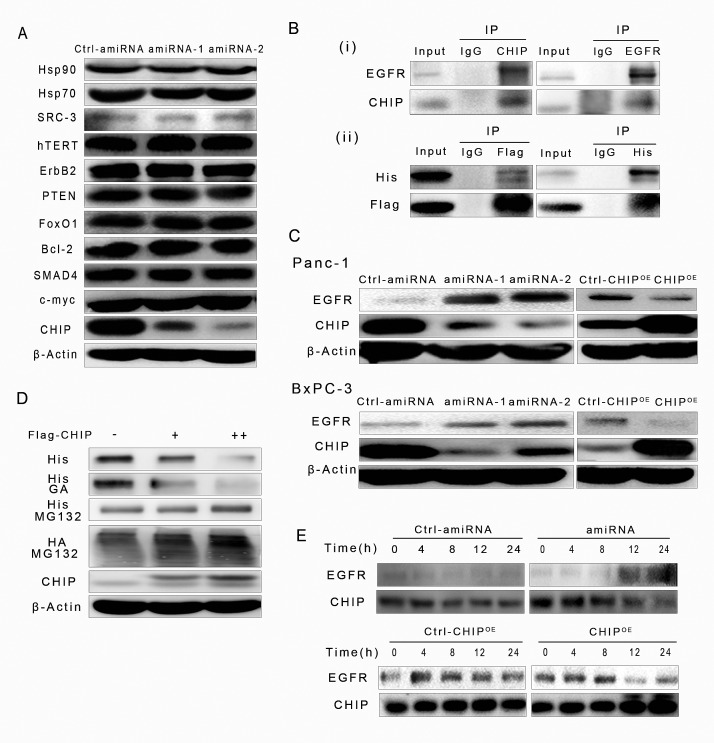
CHIP promotes EGFR ubiquitination for degradation through the ubiquitination/proteasome pathway (A) The levels of proteins that were degraded by CHIP in other types of cells were determined by immunoblotting. (B) CHIP interacts with EGFR in an endogenous or exogenous way. (i)BxPC-3 cells were pretreated with MG132 (5 μM) for 6 h, and endogenous CHIP-EGFR interaction was examined by immunoprecipitation with CHIP or EGFR antibody. (ii)BxPC-3 cells were transfected with His-EGFR and Flag-CHIP, MG132 were used after 48 h of transfection, and two exogenous protein interactions were determined by His or Flag antibody. (C) CHIP promotes EGFR degradation in Panc-1 and BxPC-3 cells. The levels of EGFR were determined after cells were infected with scrambled controls, CHIP amiRNA or CHIP overexpression(CHIP^OE^) lentiviruses. (D) CHIP enhances EGFR degradation in concentration and ubiquitination/proteasome dependent ways. BxPC-3 cells were co-transfected with HA-ubiquitin, His-EGFR and Flag-CHIP plasmids (0, 1 μg, 2 μg per well in a six-well plate). The levels of His-EGFR were determined by immunoblotting, using antibody against His-tag. Wells in another six-well plate were treated with geldanamycin (GA,1 μM) or MG132 (5 μM) for 6 h after 48 h co-transfection. The levels of His-EGFR and HA-ubiquitin were detected with anti-His or anti-HA antibody. (E) CHIP enhances EGFR degradation in a time-dependent manner. BxPC-3 cells were transfected with control,CHIP^OE^ or CHIP amiRNA for 0, 4, 8, 12, or 24 h. The levels of EGFR were determined by immunoblotting.

CHIP is a U-box E3 ubiquitin ligase that can degrade many proteins that are related to tumor progression. We compared the levels of several tumor-related proteins in control and CHIP knockdown BxPC-3 cells by immunoblotting. We did not observe a correlation between the expression of SRC-3, ErbB-2, hTERT, PTEN, FoxO1, Bcl-2, SMAD4, c-myc, Hsp70, Hsp90 and CHIP expression in BxPC-3 cells (Figure 1A), this result could be explained by feedback of complicated signaling network in different tumor environments. Given that EGFR protein is a client of Hsp90 and is also controlled by the ubiqutination/proteasome system, we hypothesized that CHIP could be involved in the modulation of the EGFR protein level in pancreatic cancer. We first investigated the possibility that CHIP physically associates with EGFR in an endogenous or exogenous way after the presence of the proteasome inhibitor MG132. Immunoprecipitation and immunoblot demonstrated that endogenous EGFR and CHIP interact with each other in BxPC-3 cells (Figure 1Bi); the His-EGFR and Flag-CHIP that were both expressed after plasmid transfection in BxPC-3 cells can also interact with each other (Figure 1Bii). Then, we examined whether the amount of CHIP is involved in regulating the stability of EGFR protein. We tested the levels of EGFR and CHIP in stable CHIP knockdown (amiRNA) or in up-regulation (CHIP^OE^) cells, including Panc-1 and BxPC-3. CHIP knockdown resulted in an up-regulation of the steady-state levels of EGFR protein, whereas the levels of EGFR were significantly lower in CHIP^OE^ cells compared to the control cells (Figure 1C). In the concentration-dependent experiment, our results showed that when the expression levels of exogenous Flag-CHIP increased, the levels of His-EGFR correspondingly decreased in BxPC-3 cells. This effect could be significantly accelerated by the Hsp90 inhibitor geldanamycin (GA). On the other hand, the expression of His-EGFR did not change much after treated with MG132, moreover, the levels of EGFR that were associated with HA-ubiqutin gradually increased (Figure 1D). In the time-dependent experiment, we demonstrated that the turnover rate of EGFR increased in CHIP^OE^ BxPC-3 cells and decreased in CHIP knockdown BxPC-3 cells compared with that in the control cells (Figure 1E). These results indicate that CHIP can associate with EGFR, recruit ubiquitin to its target protein, transfer EGFR to the proteasome and induce its degradation in pancreatic cancer cells.

**Figure 2:**
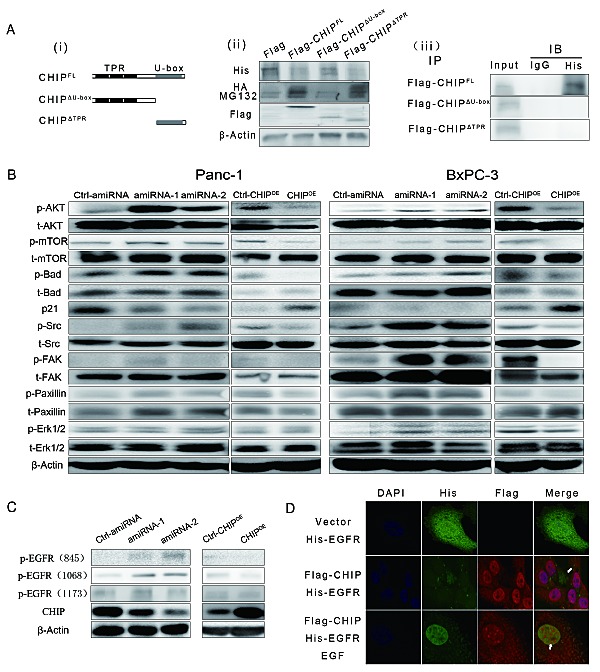
(A) (i)Two main functional domains of CHIP are illustrated schematically. (ii) The U-box domain of CHIP is required for degradation of EGFR. BxPC-3 cells were co-transfected with HA-ubiquitin, His-EGFR and Flag tagged CHIP-full length (CHIP^FL^) or its Flag tagged domains (CHIP^ΔU-box^ for CHIP protein without a U-box domain, CHIP^ΔTPR^ for CHIP protein without a TPR domain) for 48 h. Then, the cells were treated with or without MG132 (5 μM). EGFR and ubiquitin were detected by immunoblotting with anti-His or anti-HA antibody. Anti-Flag antibody was used to test the expression of CHIP^FL^ or its domains. (iii) CHIP^FL^ is required to interact with EGFR. BxPC-3 cells were co-transfected with His-EGFR and Flag-CHIP^FL^, Flag-CHIP^ΔU-box^ or Flag-CHIP^ΔTPR^ for 48 h; then, the cells were treated with MG132 (5 μM). CHIP or its domains were combined by anti-Flag antibody (IP) and immunoprecipitated by A/G agarose beads. Immunoblotting (IB) using anti-His antibody was performed to determine the exogenous expression of EGFR. (B) The downstream pathways of EGFR are regulated by CHIP in Panc-1 and BxPC-3 cells. The lysate of stable CHIP knockdown cells or CHIP^OE^ cells were used to determine the expression of AKT/mTOR, Src/FAK/paxillin,MAPK pathways by different antibodies. In the figure, p- represents phosphorylated, and t- represents total. (C) CHIP down-regulates phosphorylation of Tyr845 and Tyr 1068 of EGFR in Panc-1 and BxPC-3 cells. (D) CHIP is co-localized with EGFR in Bxpc-3 cells and attenuates the expression of EGFR. BxPC-3 cells were transfected with vector or Flag-CHIP plasmid combined with His-EGFR plasmid. After 48 h, the cells were treated with or without EGF (50 ng/mL for 30 min) and then were stained with anti-His or anti-Flag antibody. The white arrows indicate that EGFR was present or absent due to CHIP under- or over-expression in the cytoplasm or nucleus.

To determine which part of CHIP is required for the binding and down-regulation of EGFR, we created two plasmids that express the different domains of CHIP: Flag-CHIP^ΔU-box^, which expresses the TPR plus charged domain of CHIP, and Flag-CHIP^ΔTPR^, which expresses the U-box domain (Figure 2Ai). We found that CHIP^FL^ as well as CHIP^ΔTPR^ could down-regulate the expression levels of exogenous His-EGFR, while the levels of His-EGFR did not change after CHIP^ΔU-box^ transfection compared to the control. On the other hand, in BxPC-3 cells that address MG132, the ubiquitins increased significantly at the location of approximately 175KDa, which is the molecular weight of EGFR after the transfection of CHIP^FL^ or CHIP^ΔTPR^ (Figure 2Aii). At the same time, the co-immunoprecipitation (Co-IP) assay was performed to test the binding site of CHIP with EGFR. His-EGFR is precipitated following the Flag-CHIP^FL^ protein, while His-EGFR could not been pulled out by either of the two truncations (Figure 2Aiii). These results raised the possibility that the full CHIP length rather than its truncations is needed for combination with EGFR, the U-box domain of CHIP can add ubiquitin to EGFR and induce its degradation through the proteasome.

Furthermore, we investigate whether the downstream signaling pathways of EGFR could be modulated by CHIP. We found that the levels of phosphorylated (p-)AKT, mTOR, Bad, Src, FAK, and paxillin were higher in the stable CHIP knockdown Panc-1 and BxPC-3 cells, and the levels of p-AKT, p-mTOR, p-Bad, p-Src, p-FAK, and p-paxillin significantly decreased in CHIP^OE^ cells, while the total protein did not change in the CHIP knockdown and CHIP^OE^ cells. The CHIP knockdown could also decrease the level of p21^CIP1/WAF1^. Thus, CHIP can negatively regulate PI3K/AKT/mTOR and Src/FAK/paxillin pathway activation in pancreatic cancer cells. We observed that CHIP can down-regulate the level of p-Erk1/2 in Bxpc-3 cells but not in panc-1 cells, suggesting that CHIP could regulate MAPK pathway but may be influenced by other factors (Figure 2B). We also tested the levels of different phosphorylated sites of EGFR in different expression of CHIP, we found that Tyr 845 and Tyr 1068 of EGFR were regulated by CHIP expression (Figure 2C).

We next performed immunofluorescence to detect the effect of Flag-CHIP on His-EGFR. His-EGFR was predominantly localized to the membrane and cytoplasm in BxPC-3 cells while Flag-CHIP was localized to the cytoplasm and nucleus. The expression of Flag-CHIP attenuated the His-EGFR levels. After treatment with EGF that can induce EGFR from membrane to cytoplasm and nucleus, the co-localization of EGFR and CHIP was observed in the cytoplasm, and the higher levels of Flag-CHIP were accompanied by little expression of EGFR in the nucleus (Figure 2D). These results were consistent with the EGFR-CHIP interaction detected in the immunoprecipitation assay.

### Tumor growth is inhibited by CHIP *in vitro* and *in vivo*.

**Figure 3:**
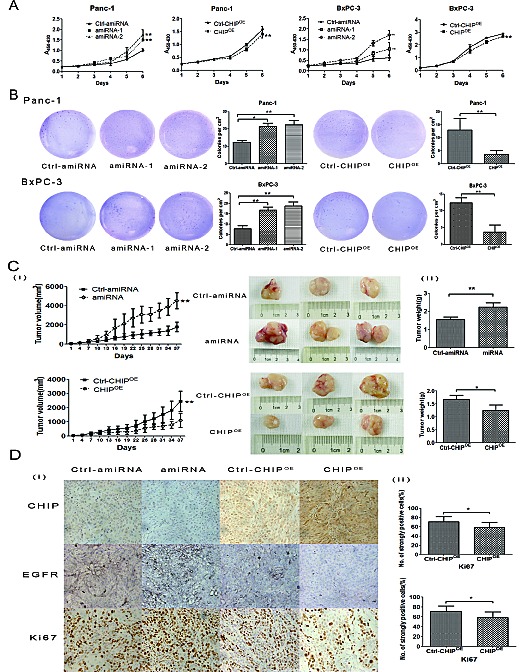
CHIP effects the growth rate of pancreatic cancer cells (A) CHIP suppresses cell growth rate. CHIP amiRNA, CHIP^OE^ and their corresponding control Panc-1 or BxPC-3 cells were grown in 96-well plates for 1, 2, 3, 4, 5 and 6 days. Cell survival was detected by CCK-8 analysis (mean±standard deviation; **P<.01). (B) CHIP suppresses anchorage-independent growth in Panc-1 and BxPC-3 cells. Stable CHIP knockdown or CHIP^OE^ cells were plated in a 6-well plate that contained soft agar. After incubation for 21 days, colonies were photographed and counted under the microscope (mean±standard deviation; *P<.05, **P<.01). (C)The stable CHIP knockdown or CHIP^OE^ cells and their control BxPC-3 cells were subcutaneously injected into nude mice. Thirty-seven days after the injections, the mice were sacrificed, and tumor tissues were collected. The left panel shows tumor growth curves in nude mice; the middle and right panel shows the size and weight of the tumors after 37 days (mean±standard deviation; *P<.05, **P<.01). (D) CHIP^OE^ decreases, but knockdown CHIP enhances the expression of EGFR and Ki67. Sections of tumors from injected nude mice were stained with CHIP, EGFR and Ki67 antibodies by immunohistochemistry (magnification ×100). The right panel shows the percentage of strongly Ki67 stained tumor cells (mean±standard deviation; *P<.05).

To examine the role of CHIP on the growth rate of pancreatic cancer cells, we performed a cell proliferation assay. Our results indicated that the CHIP knockdown in Panc-1 cells increased the ability for growth compared with negative control cells; in agreement with this finding, CHIP overexpression suppressed cell growth compared with the corresponding control. Similar results were confirmed in BxPC-3 cells (Figure 3A). In the soft agar colony formation assay, there were fewer colonies formed in the CHIP^OE^ cells, and the knockdown of CHIP significantly increased the number of colonies compared with the control cells (Figure 3B).

To address the anti-tumorigenicity of CHIP on pancreatic cancer cells in vivo, we used BxPC-3 stable CHIP knockdown or CHIP^OE^ cells in a nude mouse xenograft model. Tumor growth was significantly promoted in nude mice injected with CHIP knockdown cells compared with control mice (*P*<.01), while little tumor growth was observed in the CHIP^OE^ group compared with the control group (*P*<.01) (Figure 3C). To further determine whether CHIP decreases the EGFR expression and inhibits tumor growth, we performed immunohistochemistry to detect the expression of CHIP, EGFR and Ki67 in nude mice tumor tissues. Histological examination revealed that CHIP is only distributed in the nucleus of the CHIP knockdown cells. CHIP protein labeling was noted in a cytoplastic and nuclear distribution in the control group, and the intensity of CHIP labeling was stronger in cytoplasm and nucleus in CHIP^OE^ cells. EGFR protein was shown to be positive in a membranous distribution. EGFR expression was substantially down-regulated in the CHIP^OE^ compared with the control, whereas CHIP knockdown tumor tissues showed an up-regulated expression of EGFR in the membranes. The Ki-67 protein was mainly stained in the nucleus. The percentage of cells that were strongly labeled with the Ki-67 antibody was higher in the CHIP knockdown group compared with the control group (*P*=.021), while the percentage of Ki67 strongly positive cells decreased with an increase in the CHIP expression (*P*=.026) (Figure 3D). These results suggest that CHIP suppresses tumor progression by the inhibition of EGFR expression *in vivo*.

### CHIP enhances the sensitivity of erlotinib on apoptosis of pancreatic cancer *in vitro* and *in vivo*

**Figure 4:**
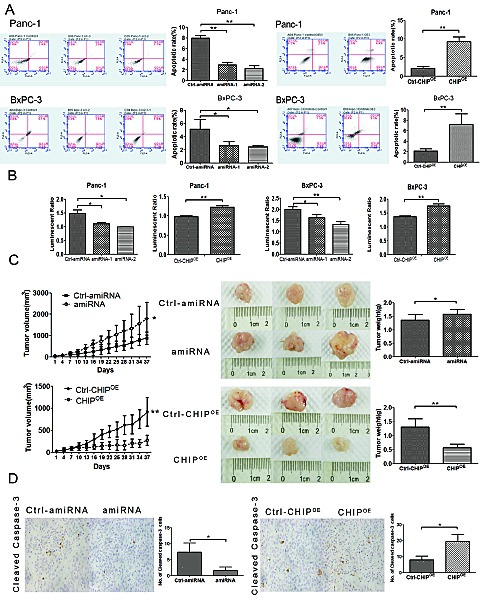
CHIP enhances the sensitivity of erlotinib on apoptosis and tumor growth (A) CHIP enhances the apoptotic rate measured by FACS assay after cells were treated with erlotinib. The stable CHIP knockdown or CHIP^OE^ with their control cells were treated with erlotinib for 1 day (Panc-1,20μM; BxPC-3,1μM). The cells were stained with Annexin V-PE and 7-AAD, and the apoptotic rate was assessed by FACS (mean±standard deviation; *P<.05, **P<.01). (B) CHIP enhances the apoptotic rate determined by Caspase 3/7 assay after cells were treated with erlotinib. Caspase-3/7 activity was determined using the Caspase-Glo 3/7 assay kit after 1 day of treatment with erlotinib (mean±standard deviation; *P<.05, **P<.01). (C) CHIP enhances erlotinib-induced tumor growth inhibition. The stable CHIP knockdown or CHIP^OE^ cells and their control (Ctrl) BxPC-3 cells were subcutaneously injected into nude mice. The mice were treated daily with 50 mg/kg erlotinib beginning on day 7, and the mice were killed and tumor tissues were collected after 30 days of drug treatment. The left panel shows tumor growth curves in nude mice; the middle and right panel indicates the size and weight of the tumors after erlotinib treatment (mean±standard deviation; *P<.05, **P<.01). (D) CHIP enhances erlotinib-induced tumor apoptosis. Sections of tumors from injected nude mice were stained with cleaved caspase-3 antibody by immunohistochemistry. The numbers of positive stained cells were counted (magnification ×100), (mean±standard deviation; *P<.05).

Because erlotinb is a tyrosine kinase inhibitor that targets EGFR and CHIP might target EGFR for degradation, we sought to investigate the synergistic effect of CHIP and erlotinb on tumor apoptosis. We first examined the apoptotic rate of pancreatic cancer cells treated with erlotinib under different CHIP levels. Flow cytometric analysis showed a higher induction of apoptosis in CHIP^OE^ Panc-1 and BxPC-3 cells compared with the control cells. In line with this finding, the apoptotic rate decreased significantly in CHIP knockdown cells (Figure 4A). To further validate our data, we next checked the activity of caspase3/7 after treatment with erlotinib under different CHIP expressions. CHIP knockdown led to a decreased activation of caspase 3/7, while an increased activation of the caspase 3/7 was observed in CHIP^OE^ cells after they were exposed to erlotinib (Figure 4B).

To confirm the effect of CHIP on erlotinib-induced tumor growth inhibition and apoptosis *in vivo*, a xenotransplantation assay on nude mice was performed. After 30 days of treatment with erlotinib, the tumor volume of BxPC-3 xenografts in the CHIP knockdown group was increased compared with the control tumors (P=.034). In contrast, the tumor growth ability in mice injected with CHIP^OE^ cells was significantly abrogated (*P*<.01) (Figure 4C). Immunohistochemical analysis of treated tumor xenografts of BxPC-3 cells were measured using the cleaved caspase-3 antibody. CHIP knockdown showed a decrease in the numbers of apoptotic cells, while cleaved caspases-3 labeling cells increased sharply in tissues that overexpressed CHIP (Figure 4D). These observations demonstrate that CHIP can enhance the ability of erlotinib on tumor growth inhibition and apoptosis *in vitro* and *in vivo*.

### CHIP attenuates migration and invasion of pancreatic cancer cells *in vitro* and *in vivo*

**Figure 5:**
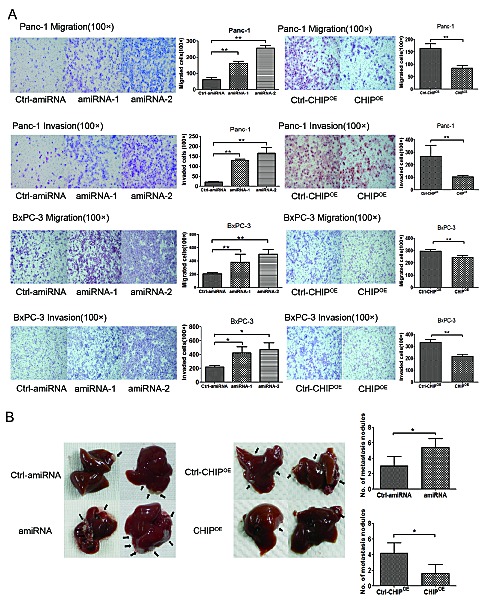
CHIP inhibits the migration and invasion of pancreatic cancer cells (A) CHIP inhibits the ability of migration and invasion of cells measured by chamber assay. Panc-1 or Bxcp-3 stable CHIP knockdown or CHIP^OE^ cells were added to the upper portion of a chamber that was coated with or without ECM. After 48 h, cells on the lower side of the membrane were fixed, stained with HE and counted under the microscope (magnification ×100. mean±standard deviation; *P<.05, **P<.01). (B) CHIP inhibits tumor liver metastases in mice. Bxcp-3 CHIP knockdown or CHIP^OE^ with its control.Cells were injected into the spleens of mice. After 6 weeks, the mice were killed and the livers were collected. The number of metastatic foci on the liver surface was counted (mean±standard deviation; *P<.05).

To examine the roles of CHIP in the invasion and migration potential of pancreatic cells, we performed a Transwell assay. The invasiveness of CHIP knockdown cells was significantly increased compared with the control Panc-1 cells, whereas CHIP^OE^ decreased the number of cells that penetrated the ECM-coated membrane. Similar results were confirmed in BxPC-3 cells. In line with this finding, the migration abilities of two pancreatic cancer cells were enhanced after CHIP knockdown, while CHIP^OE^ reduced the number of cells that penetrated the 8 μm pore size membrane compared with the control group (Figure 5A).

To investigate whether the CHIP level changes in BxPC-3 cells regulate *in vivo* metastatic activity, we injected control and CHIP knockdown or CHIP^OE^ cells into the spleen of nude mice. Liver metastasis was enhanced in the mice that were given the CHIP knockdown cells (*P*<.01). In contrast, CHIP^OE^ in the BxPC-3 cells reduced the liver metastasis compared to the control cells (*P*<.01) (Figure 5B). Our observations show that CHIP attenuates pancreatic cell invasion and migration *in vitro* and *in vivo*.

### The expression of CHIP in pancreatic cancer tissues or sera and its clinical significance.

**Figure 6:**
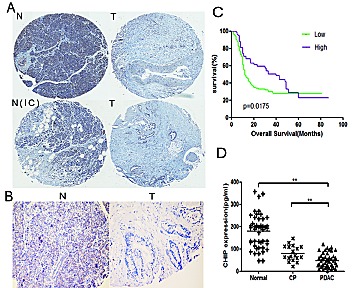
The levels of CHIP are decreased in human pancreatic cancer tissues and sera (A,B) The pancreatic tissues were stained by immunohistochemistry with CHIP antibody. T represents tumor tissues; N represents the adjacent normal tissue; and N(IC) represents the adjacent normal tissues that are infiltrated with inflammatory cells (A,magnification ×40; B,magnification ×100). (C) Kaplan-Meier curves that depict the overall survival according to the CHIP expression in patients with pancreatic cancer (n=202, p=0.0175). Low, CHIP low expression group; High, CHIP high expression group. (D) Individual serum levels of CHIP in normal controls (Normal), patients with chronic pancreatitis (CP),and pancreatic adenocarcinoma patients (PDAC) (mean±standard deviation; **P<.01).

Samples of pancreatic cancer tumors and adjacent normal tissues were obtained from 225 patients. The protein levels of CHIP in human pancreatic cancer tissues were examined by immunohistochemistry. The results showed that CHIP protein was localized mainly in the cytoplasm of pancreatic cancer cells and adjacent non-cancerous cells (Figure 6A, B). The level of CHIP expression was decreased in pancreatic cancer tissues compared with corresponding non-cancerous pancreatic tissues (P=.038) (Table I). In addition, the expression of CHIP in pancreatic cancer tissues was significantly reduced compared to matched normal tissues without inflammatory cellular infiltration (p<.01) (Table II), while there was no significant difference between pancreatic cancer tissues and paired non-cancerous tissues infiltrated with inflammatory cells (P=0.558) (Table III), which suggests that inflammation could affect the expression of CHIP in pancreatic tissues.

**Table I:** The expression of CHIP in the pancreatic cancer tissues and their adjacent normal tissues(χ2 test)

	CHIP expression		P value
	Low	High	
			0.038
Normal tissues	107	118	
Tumor tissues	129	96	

**Table II:** The expression of CHIP in the pancreatic cancer tissues and their adjacent normal tissues without inflammatory cells infiltration(χ2 test)

	CHIP expression		P value
	Low	High	
			0.001
Normal tissues (without inflammatory cells)	45	82	
Tumor tissues	71	56	

**Table III:** The expression of CHIP in the pancreatic cancer tissues and their adjacent normal tissues with inflammatory cells infiltration(χ2 test)

	CHIP expression		P value
	Low	High	
			0.558
Normal tissues (with inflammatory cells)	62	36	
Tumor tissues	58	40	

In the 202 patients with follow-up, CHIP expression was negatively correlated with tumor differentiation (P=.036). However, CHIP expression was not significantly correlated with patient age, gender, tumor size, TNM stage or perineural invasion (Table IV). Kaplan-Meier analysis revealed that the 1-year overall survival rates for the patients with low and high CHIP expression were 49.1% and 70.8%, respectively. The median survival time of the patients with low CHIP expression was 12 months while a high expression of CHIP correlated with a median survival time of 40 months (Table V). Lower CHIP staining was significantly correlated with a poorer overall survival of pancreatic cancer patients (P=.0175) (Figure 6C). Multivariate Cox regression analysis that included gender, tumor differentiation, N-stage, M-stage, perineural invasion and CHIP expression showed significance in the univariate survival analyses. CHIP expression was an independent prognostic factor (P=.001). The high expression of CHIP in histological sections had a statistically significant hazard ratio of 0.515 (95%CI 0.347 to 0.765) (Table VI).

**Table IV:** Correlations between expression levels of CHIP and clinicopathological features

Variables	No. of patients	CHIP expression		P value
		Low	High	
Age				0.728
<65	135	86	49	
≥65	67	41	26	
Gender				0.274
Male	123	81	42	
Female	79	46	33	
Tumor location				0.912
Head	131	82	49	
Body/tail	71	45	26	
Tumor diameter(cm)				0.439
≤3	66	39	27	
>3	136	88	48	
Histological grade				0.036*
Grade 1	12	6	6	
Grade 2	128	89	39	
Grade 3	62	32	30	
Pathological T stage				0.179
T1/T2	133	88	45	
T3/T4	69	39	30	
Lymph node metastasis				0.356
N0	110	66	44	
N1/2/3	92	61	31	
Distant metastasis				0.712*
M0	195	123	72	
M1	7	4	3	
TNM stage				0.646
I/II	188	119	69	
III/IV	14	8	6	
Perineural invasion				0.583
No	142	91	51	
Yes	60	36	24	

* two sided Fisher's exact tests.

**Table V:** Univariate analysis of the association of prognosis with clinicopahtological parameters and CHIP expression in 202 patients with pancreatic adenocarcinoma

Variables	No. of patients	Overall survival(Months)		1-year survival rates	P value
		median±SD	95% CI		
Age					0.446
<65	135	15±2	11-19	55.9%	
≥65	67	20±9	3-37	59.5%	
Gender					0.007
Male	123	13±1	10-16	53.1%	
Female	79	43±14	16-70	63.2%	
Tumor location					0.557
Head	131	18±4	11-25	60.7%	
Body/tail	71	15±3	10-20	50.2%	
Histological grade					0.004
Grade 1	12	-	-	100%	
Grade 2	127	17±4	8-26	58.6%	
Grade 3	61	12±1	9-15	47.4%	
T stage					0.421
T1/T2	133	17±3	12-22	57.4%	
T3/T4	69	17±4	10-24	57.1%	
Lymph node metastasis					<0.001
N0	110	33±8	17-49	72.8%	
N1/2/3	92	11±0	10-12	37.6%	
Distant metastasis					0.043
M0	195	17±2	13-21	57.7%	
M1	7	7±1	4-10	42.9%	
TNM stage					0.336
I/II	188	17±2	12-22	57%	
III/IV	14	17±8	1-33	61.5%	
Perineural invasion					0.034
No	142	19±6	8-30	61.3%	
Yes	60	12±1	10-14	47.8%	
CHIP expression					0.0175
Low	127	12±1	9-15	49.1%	
High	75	40±6	28-52	70.8%	

**Table VI:** Multivariate Cox regression analysis of CHIP and clinical variables for overall survival

Variables*	Hazard ratio	95% confidence interval	P value
Gender	0.756	0.514 to 1.112	0.156
Histological grade	2.263	1.593 to 3.213	<0.001
Lymph node metastasis	2.678	1.862 to 3.852	<0.001
Distant metastasis	2.061	0.795 to 5.348	0.137
Perineural invasion	1.624	1.124 to 2.347	0.01
CHIP expression	0.515	0.347 to 0.765	0.001

* Coding of variables: gender was coded as 1, male and 2, female; histological grade was coded as 1 Grade1, 2 Grade 2 and 3 Grade 3; N stage was coded as 1,N0 and 2,N1/2/3; M stage was coded as 1,M1 and 2,M2;CHIP expression was coded as 1, low and 2, high.

To explore the expression of CHIP in the serum we detected CHIP levels in 47 sera samples from patients who had pancreatic cancer, as well as in age- and sex-matched normal subjects (n=47) and in 18 patients who had chronic pancreatitis. As a result, the median serum CHIP level was significantly lower in the patients who had pancreatic adenocarcinoma compared with healthy volunteers (P<.001) and chronic pancreatitis patients (P=.001) (Figure 6D). The median CHIP level was 48.26 pg/ml for patients who had pancreatic adenocarcinoma; for patients with chronic pancreatitis, the median was 80.27 pg/ml; and for normal controls, the median was 179.99 pg/ml. We also measured the relationship between CHIP expression in serum and various clinicopathological parameters in pancreatic cancer patients, The expression of CHIP had an inverse correlation with distant metastasis (P=.01) (Table VII), but the serum levels of CHIP were not correlated with the survival time (P=.602) (Table VIII). These results indicated that the levels of CHIP were also decreased in pancreatic cancer sera, and the expression of CHIP could be a tool to determine whether distant metastases occur in pancreatic cancer patients.

**Table VII:** Correlations between serous expression levels of CHIP and clinicopathological features

Variables	No. of patients	CHIP expression		P value
		Low	High	
Age				0.936
<65	29	19	10	
≥65	18	12	6	
Gender				>0.598
Male	26	13	8	
Female	21	18	8	
Tumor location				0.337
Head	28	20	8	
Body/tail	19	11	8	
Histological grade				1*
Grade 1/2	35	23	12	
Grade 3	12	8	4	
T stage				1*
T1/T2	12	8	4	
T3/T4	35	23	12	
Lymph node metastasis				0.719
-	16	10	6	
+	31	21	10	
Distant metastasis				0.01*
-	37	21	16	
+	10	10	0	
TNM stage	>			0.769
I/II	28	18	10	
III/IV	19	13	6	

* two sided Fisher's exact tests.

**Table VIII:** Univariate analysis of the association of prognosis with clinicopahtological parameters and serous CHIP expression in patients with pancreatic adenocarcinoma

Variables	No. of patients	Overall survival(Months)		1-year survival rates	P value
		median±SD	95% CI		
Age					0.825
<65	29	16±3	11-21	58.6%	
≥65	18	18±3	12-24	65.8%	
Gender					0.801
Male	21	18±1	15-21	61.5%	
Female	26	16±3	10-22	61.5%	
Tumor location					0.863
Head	28	18±5	9-27	66.1%	
Body/tail	19	16±2	13-19	82.3%	
Histological grade					0.309
Grade 1/2	35	18±1	17-19	59.5%	
Grade 3	12	15±2	12-18	66.7%	
T stage					0.771
T1/T2	12	17±4	10-24	66.7%	
T3/T4	35	18±2	15-21	59.5%	
Lymph node metastasis					0.021
N0	16	-	-	87.5%	
N1/2/3	31	12±4	4-20	47.8%	
Distant metastasis					<0.001
M0	37	21±5	12-30	70%	
M1	10	6±1	4-8	30%	
TNM stage					<0.001
I/II	27	30±6	18-42	88.7%	
III/IV	20	8±1	5-11	25%	
Resection					0.004
No	28	21±7	7-34	78.3%	
Yes	19	10±3	4-16	36.8%	
Serous CHIP expression					0.602
Low	31	18±3	12-24	57.5%	
High	16	16±3	11-21	68.8%	

## DISCUSSION

Pancreatic cancer (PC) is one of the most aggressive tumors with an extremely poor prognosis. Overexpression of EGFR and its persistent activation has been reported to contribute to tumor aggressiveness and chemoresistance in pancreatic cancer[17]. The degradation of EGFR protein relies on c-Cbl protein, which is an E3 ubiquitin ligase that recruits ubiquitin to its substrate for degradation [18]. Until now, whether there are other ubiquitin ligases that can induce EGFR degradation remains elusive. CHIP is an E3 ubiquitin ligase that serves as a bridge to transfer the protein from the chaperone Hsp90 to the ubiquitin-proteasome system [19]. Previous data suggested that CHIP could induce ErbB2 ubiquitination and degradation in breast cancer cells [10]. EGFR, which shares close structural homology with ErbB2, has been shown to be a client of Hsp90 and has maintained stability in many cancer cells[20]. Thus, CHIP could theoretically function as a new ubiquitin ligase that can target EGFR for degradation. Our results confirmed that CHIP could interact with EGFR in pancreatic cancer cells. In addition, CHIP also recruited ubiquitin to EGFR and transferred its target to the proteasome for degradation. Furthermore, CHIP accelerated the degradation of EGFR when the cells were treated with the Hsp90 inhibitor geldanamycin. This result is similar to the function of CHIP on ErbB2. Xu W *et al.* reported that both the CHIP and GA decrease the amount of ErbB2 associated with Hsp90, and the CHIP expression shortens the half-life of the ErbB2 protein[21].

Our study showed that CHIP induced the degradation of EGFR and inactivated its downstream PI3K/AKT pathway as well as the Src/FAK/paxillin pathways. The activation of two pathways was reported to be involved in proliferation, apoptosis, invasion and migration in pancreatic cancer cells [22, 23]. Furthermore, MAPK pathway could also be influenced by the expression of CHIP that were observed in Bxpce-3 cells, but not in Panc-1 cells that contain mutant K-ras gene,the reason may be that MAPK signaling pathway were constitutively activated by mutation of K-ras that exhibited little response to EGFR regulation in Panc-1 cells[24]. In addition, we observed that phosphorylation of Tyr845 and Tyr1068 of EGFR was regulated by CHIP, Tyr845 of EGFR could be associated with Src and is involved in tumor malignancy or resistance to EGFR-targeted therapy [25, 26]. Phosphorylation site 1068 of EGFR forms a complex with Grb2 and increases mitogen-activated protein kinase activation [27]. In accordance with this finding, we found that CHIP knockdown enhanced the proliferation, colony formation, invasion and migration of Panc-1 and BxPC-3 pancreatic cancer cell lines *in vitro*, while CHIP^OE^ obtained the opposite results. Moreover, tumor growth in the mouse xenografts was significantly enhanced after the injection of CHIP knockdown BxPC-3 cells, whereas the tumor growth rate was inhibited after CHIP^OE^ cells were injected. The expression of CHIP also inhibited the number of liver metastases in nude mice. All of these results indicated that CHIP could act as a tumor suppressor that prohibits tumorigenesis and tumor metastasis in pancreatic cancer. The function of CHIP in pancreatic cancer is consistent with its role in other malignant cells. Jang KW and colleagues reported that CHIP destabilizes the Met receptor and inhibits tumor growth, motility and invasion in lung cancer cells [11]. Kajiro M *et al*. observed that CHIP suppresses tumor progression by direct degradation of the oncogene SRC-3 in breast cancer cells[12]. Wang S *et al*. reported that CHIP can down-regulate the subunit protein of NF-κB and inhibit gastric tumorigenesis and angiogenesis [13]. On the other hand, CHIP has been shown to enhance tumor proliferation by increasing the expression of survivin protein in human glioma cancer cells [28], which indicates that CHIP might play different roles in different human cancers.

In pancreatic adenocarcinoma, the EGFR tyrosine kinase domain is highly conserved, which indicates that this tumor is responsive to EGFR target therapy. Elotinib is an oral EGFR tyrosine kinase inhibitor that can inhibit the growth and metastasis of human pancreatic tumor xenografts [29]. Morgan *et al*. reported that phosphorylation of Tyr1173 of EGFR is the target of erlotinib [30],while we observed that phosphorylation of Tyr845 and Tyr1068 of EGFR could be regulated by CHIP,thus the multitarget treatment may explain the phenomena that CHIP enhanced the efficacy of erlotinib on pancreatic tumor growth and apoptosis. More importantly, CHIP could also increase the apoptotic rate induced by erlotinib in Panc-1 cells that present K-ras mutations; mutant K-ras has been viewed as a potential molecular predictor of responses to EGFR inhibition [31]. For these reasons, we thought that CHIP might be a potential treatment target for pancreatic cancer.

In the present study, we observed that pancreatic cancer tumors exhibited a relatively lower level of CHIP expression compared with adjacent normal tissues. The expression of CHIP was correlated with tumor differentiation. Moreover, statistical analysis indicated that the reduced expression of CHIP was negatively associated with survival in pancreatic cancer patients and it was one of the independent risk factors that affected the prognosis in pancreatic cancer patients. To be consistent with our results, CHIP levels have been proven to be negatively correlated with the malignancy of gastric tumor tissues [13], whereas studies on other digestive tumors obtained the opposite results. In a study on esophageal squamous cell carcinoma (ESCC), the level of CHIP was higher in the metastatic lymph nodes compared with the primary tumors as well as in the normal esophageal epithelia. The high level of CHIP in metastatic lymph nodes was an independent prognostic factor in ESCC [32]. Liang ZL et al. reported that the high expression of CHIP indicated a significantly worse prognosis in gallbladder carcinoma patients[33]. All of these results indicate that the pathogenic mechanisms of CHIP expression in human gastrointestinal cancer are different and still require further investigation.

Until now, there were no experiments that measured the expression of CHIP in cancer patients' sera. Our tests suggested that CHIP expression was lower in pancreatic cancer compared with healthy controls and chronic pancreatitis. The expression of CHIP was also lower in chronic pancreatitis, which was coincident with the immunohistochemical protein staining in the normal tissues infiltrated with inflammatory cells. This result indicates that inflammation could affect the expression of CHIP in pancreatic tissue and serum.

In conclusion, our study demonstrated that CHIP serves as a novel EGFR-mediated E3 ligase and attenuates the downstream EGFR signaling pathways in pancreatic cancer cells. Also, CHIP acts as a tumor suppressor by inhibiting cell proliferation, anchorage-independent growth, invasion and migration, as well as enhancing cell apoptosis induced by erlotinib *in vitro* and *in vivo*. We also showed that there is lower expression of CHIP in pancreatic cancer tissues and sera; the negative relationship between CHIP expression and tumor malignancy indicates that CHIP may serve as a potential treatment target of pancreatic cancer.

## MATERIAL AND METHODS

### Cell Lines and Reagents

The human pancreatic cancer cell lines Panc-1 and BxPC-3 were type gifts from Dr. Freiss H (University of Heidelberg, Heidelberg, Germany). The cells were cultured in Dulbecco's modified Eagle's medium (DMEM) or RPMI-1640 medium (Hyclone, Utah, USA), supplemented with 10% fetal bovine serum (FBS, Hyclone), 1% penicillin and streptomycin in a humidified incubator of 5% CO_2_ at 37°C. Extracellular matrix (ECM) was purchased from Sigma-Aldrich (Shanghai, China). MG132 was provided by Selleckchem (Houston, USA). EGF was procured from Invitrogen (Shanghai,China). Erlotinib (Tarceva) was obtained from Roche (Basel,Switzerland) and dissolved in DMSO as a stock solution at 1 mM concentration for the cell testing or in 0.5% CMC-Na for mouse intragastric administration. Antibodies and their sources were as follows: anti-CHIP antibody (Santa Cruz,California,USA); anti-EGFR antibody and anti-phosphorylated EGFR (Tyr845, Tyr1068, Tyr1173) antibody (Cell Signaling,Massachusettes,USA); anti-AKT antibody and anti-phosphorylated AKT (Ser473) antibody (Cell Signaling); anti-mTOR antibody and anti-phosphorylated mTOR (Ser2448) antibody (Cell Signaling); anti BAD antibody and anti-phosphorylated BAD (Ser136) antibody (Cell Signaling); anti-p21 antibody (Cell Signaling); anti-Src antibody and anti-phosphorylated Src (Tyr416) antibody (Cell Signaling); anti-FAK antibody and anti-phosphorylated FAK (Tyr 925) antibody (Cell Signaling); anti-paxillin antibody and anti-phosphorylated paxillin (Tyr118) antibody (Cell Signaling); anti-Erk1/2 antibody and anti-phosphorylated Erk1/2 (Thr202/Tyr204) antibody (Cell Signaling); anti-His antibody (Santa Cruz); anti-Flag antibody (Santa Cruz); anti ki67 antibody (Abcam,Cambridge,UK); and anti-Cleaved Caspase-3 antibody (Cell Signaling).

### Plasmids or Lentiviruses for Transfection or Infection

CHIP artificial miRNA (amiRNA) duplexes were selected for CHIP silencing; the sequences that were synthesized are the following: 5'-TGCTGAGAAGTGCGCCTTCACAGACTGTTTTGGCCACTGACTGACAGTCTGTGGGCGCACTTCT-3'(sense), 5'-CCTGAGAAGTGCGCCCACAGACTGTCAGTCAGTGGCCAAAACAGTCTGTGAAGGCGCACTTCTC-3'(antisense), and a loop sequence was used to separate the complementary domains. Scrambled sequences were used as control. miRNA duplexes were ligated to the vector pcDNA6.2 (Invitrogen) for reconstructions. The recombinant vectors encoding human CHIP were constructed by PCR-based amplification and were then subcloned into the pcDNA3.1 expression vector (Invitrogen). Vector encoding of HA-tagged Ubiquitin, Flag-tagged CHIP full length(CHIP^FL^), Flag-tagged CHIP^ΔTPR^, Flag-tagged CHIP^ΔU-box^, and His-tagged EGFR were constructed and inserted into pReceiver plasmids (GeneCopoeia, Guangzhou, China). For transient transfection, the pancreatic cancer cells were prepared to 70-80% confluence in 6-well plates and were transfected with plasmids using Lipofectamine 2000 (Invitrogen) following the manufacturer's instructions. Two days after transfection, cancer cells were used for subsequent experiments.

The recombinant lentiviruses were packaged using the pLenti6.2 miR RNAi expression system for knockdown or the pLent6.31expression system for overexpression (Invitrogen). Briefly, recombinant was produced by co-transfecting 293T cells with the lentivirus amiRNA plasmid (pLenti6.2-miRNA) or overexpression plasmid (pLenti6.31-CHIP) and packaging plasmids (pLP1, pLP2 and pLP/VSVG, Invitrogen) using lipofectamine2000 transfection reagent. Panc-1 and BxPC-3 cells were infected with the lentivirus, which produced amiRNA directed against CHIP or the lentivirus overexpressing CHIP(CHIP^OE^) or lentivirus with negative control sequences (Control). The transduction efficiency was between 70% and 95%. The cells were stably screened with Blasticidin (Invitrogen) at a concentration of 10 μg/ml for Panc-1 and 9 μg/ml for BxPC-3.

### Immunoprecipitation, Gel electrophoresis, Western blot analysis

For immunoprecipitation, cells were seeded in 6-well plates and incubated with 50 μM MG132 for 6 h, to inhibit the activity of the proteasomes. The cells were then lysed in modified RIPA buffer. Cell lysates were incubated with antibody for 12 h at 4 °C on a rotating plate. The proteins were immunoprecipitated by protein A/G agarose beads (Santa Cruz, USA). Samples were resolved by SDS-PAGE and subjected to immunoblot analysis.

Pancreatic cancer cells were grown to near-confluency in 6-well culture plates. The cells were washed twice with FBS in each well and ruptured by sonication using 200 μL of RIPA lysis buffer (Millipore, USA), which contained a protease and phosphatase inhibitor cocktail (Sigma-Aldrich). Cell lysates were centrifuged at 12000 r.p.m. for 12 min. Supernatants were measured with a BCA protein assay kit (Pierce, USA) and stored at -80 °C. The protein samples (80 μg) were separated on 6-8% SDS-polyacrylamide gels and electrotransferred to polyvinylidene difluoride (PVDF) membranes. The PVDF membranes were blocked for 1 h with 0.5% Tween 20 in TBS (TBST), which contained 5% non-fat dry milk, and they were incubated with antibodies for binding to the proteins at 4 °C overnight. After washes with TBST, the membranes were incubated in peroxidase-conjugated secondary antibodies against mouse or rabbit for 1 h at room temperature. They were washed and detected using the enhanced chemiluminescence (ECL) detection system (Millipore, USA).

### Immunofluorescence Assay

BxPC-3 in the slide chambers (NUNC, Denmark) were transfected with Flag-CHIP vector and His-EGFR vector for 24 h. The cells in one chamber were treated with EGF (100 ng/ml, Invitrogen) for 1 h after transfection. The cells were fixed in methanol, blocked with 10% FBS and then incubated with mouse anti-His antibody and rabbit anti-Flag antibody. The anti-His staining was detected with FITC-conjugated goat anti-mouse antibody and Flag with Rhodamine-labeled goat anti-rabbit antibody. Nuclei were stained with DAPI. The slides were imaged with a UltraVIEW VoX-3D system (Perkin-Elmer, Massachusetts , USA). The images were merged using Volocity Demo software.

### Cell Proliferation Analysis

The cell proliferation assay was evaluated using the CCK-8 kit (Dojindo, Japan). In brief, after the CHIP knockdown or overexpression in pancreatic cancer cells was confirmed by RT-PCR and western blot, cells were seeded in flat-bottomed 96-well plates at 1000 cells per well. A CCK-8 assay was performed at the time point from day 1 to 6. After 2 hours of incubation with cell culture medium that contained CCK-8 reagent, the absorbance values at 450 nm were detected using an absorbance microplate reader (SepctraMax 190, Molecular Devices), and a wavelength of 630 nm was used as a reference.

### Colony Formation Assay

Soft agar assays were measured as follows: 1 mL base layers consisting of 0.6% agar medium was prepared in 6-well plates. Cells infected with negative control, CHIP artificial miRNA(amiRNA) for RNAi, or CHIP overexpression(CHIP^OE^) lentiviruses were suspended in 0.3% agar medium supplemented with 20% FBS and 1×DMEM. The cells were placed 5000 per well for Panc-1 and 7000 per well for BxPC-3. After 21 days of incubation, the colonies were stained with crystal violet solution, and the number of colonies was counted under the microscope.

### Apoptosis Assay

Cell apoptotic assay was performed using the Annexin V-PE and 7-AAD kit from Beckman Coulter (USA) using the manufacturer's instructions. Analysis was conducted using the Accuri C6 flow cytometer (Becton Dickinson, New Jersey USA). Apoptosis was measured using a luminescence method that quantifies caspase-3/7 activity, which was determined using the Caspase-Glo 3/7 assay kit (Promega) according to the manufacturer's protocol.

### Migration and Invasion Assay

Migration and invasion were performed in a double chamber assay (8 μm pore size, Corning). The membranes for the invasion assay were coated with diluted ECM solution. The cells were added to the upper portion of a chamber with serum-free media. Medium containing 10% FBS served as a chemoattractant in the lower chamber. After incubation for 24 h, the cells from the upper side of the membrane were scraped and removed by cotton swabs. The cells on the lower side of the filter were fixed with methanol and then stained with hematoxylin and eosin. Cells in 5 visual fields distributed over the membrane were counted.

### *In Vivo* Xenograft Experiments

A total of 20 female BALB/c nude mice that were six weeks old were obtained from the Chinese Academy of Medical Sciences (CAMS), Beijing, China and maintained under pathogen-free conditions. All of the experiments were approved by the Animal Care and Use Committee of CAMS. The mice were randomly divided into four groups. Group 1 mice received 5×10^6^ BxPC-3 CHIP knockdown cells in 200 μL of PBS subcutaneously into the right flank of each mouse. Group 2 received injections of negative control cells with the same number and volume as group 1. Group 3 received inoculations of CHIP^OE^ (5×10^6^/200μL/mouse), and group 4 received injections of negative control of CHIP^OE^ with the same concentration as group 3. Tumors were measured every three days in two dimensions with calipers. The tumor volumes were calculated by the equation Volume=1/2×length×(width)^2^.

Twenty mice were randomly divided into 4 groups and inoculated with BxPC-3 cells in the above-mentioned way. The mice were treated orally daily with 50 mg/kg erlotinib on the basis of individual weights after tumors were palpable on day 7. Tumor volumes were measured every three days. These mice were sacrificed, and the tumors were excised for further research after drug treatment for 30 days.

A total of 20 nude mice were divided into 4 groups and anesthetized with 1% chloral hydrate; 5×10^5^/20μL BxPC-3 CHIP knockdown cells or controls were injected in the spleen of each nude mouse during open laparotomy, and 5×10^5^/20μL BxPC-3 CHIP^OE^ cells or controls were injected in the spleen of each nude mouse during open laparotomy for experiments. After 6 weeks, the mice were sacrificed by decapitation under adequate anesthesia, and the number of metastatic foci on the liver surface was counted. The small nodes were determined by microscopic analysis after fixation of liver and HE stain.

### Tissue and Serum Samples

A total of 225 consecutive patients with pancreatic cancer between January 2004 and December 2011 in Peking Union Medical College Hospital were included in this study. All of the patients related to this study provided informed consent with the approval of the Committee and Research Ethics Board of the Peking Union Medical College Hospital. The diagnosis of pancreatic adenocarcinoma was based on histological confirmation from operative specimens. Exclusion criteria included patients with other organic diseases and the inability to provide informed consent.

A total of 47 serum samples were obtained from patients at the time of diagnosis of pancreatic adenocarcinoma. Sera were also obtained from 18 patients with chronic pancreatitis with a confirmed clinical diagnosis and from 47 control healthy individuals collected at Peking Union Medical College Hospital. The sera from the normal subject group were age- and sex-matched to the tumor group. The samples were processed using the same procedures, and 20 ml of blood was placed in the serum separator tubes. Samples were collected and centrifuged at 3000 rpm for 10 minutes. The serum was transferred to a 1.5 mL tube and then stored at -80°C. All of the serum samples were labeled with a unique marker to protect the confidentiality of the patient. None of the samples were thawed more than twice before the analysis.

### Immunochemistry

Immunohistochemistry was performed on paraffin-embedded sections. The tissues were fixed in 4% formaldehyde overnight and embedded in paraffin wax. Sections were deparaffined in xylol and rehydrated using graded ethanol. Antigen retrieval was performed using a high pressure method for 3 min with citrate buffer. Sections were treated with 3% H_2_O_2_ for 10 min to eliminated endogenous peroxidase. Quenched sections were incubated in non-immune serum for 20 min and then added with the appropriate dilution of each primary antibody (a 1:200 dilution of anti-EGFR antibody, a 1:200 dilution of anti-CHIP antibody, a 1:300 dilution of anti-Ki67 antibody or a 1:100 dilution of anti-leaved caspase-3 antibody) overnight at 4°C, followed by incubation with linked reagent for 30 min. For the negative control, the immunostaining processes were performed by using PBS as a substitute for the primary antibody. The antigen-antibody complex was detected by using diaminobenzidine (DAB) substrate. All of the sections were then counterstained with haematoxylin, dehydrated in a graded series of ethanols and xylol, and mounted. Slides were reviewed by light microscopy. CHIP expression in tissues was evaluated in a blinded fashion by an experienced pancreatic pathologist. Visual fields (×400 magnifications) were chosen to calculate the percentage of positively stained cells over the total number of tumor cells.

The staining proportion of the positive cells was divided into four groups: negative, 0 positive cells found; +, <30% of tumor cells observed; ++, 30%-60% of tumor cells were immunopositive; and +++, >60% of tumor cells observed. Cases with proportion scores of – and + were included in the CHIP low expression group, while those with proportion scores of 2+ and 3+ were included in the CHIP high expression group for all of the analysis.

### ELISA

An ELISA assay was performed with a human CHIP ELISA kit (CUSABIO Inc, Wuhan, China). In brief, antibody specific for human CHIP protein has been pre-coated onto a microplate. Standards and samples were pipetted into the wells, and any CHIP proteins were bound by the immobilized antibody. After removing any unbound substances, a biotin-conjugated antibody specific for CHIP is added to the wells. After washing, avidin conjugated Horseradish Peroxidase (HRP) was added to the wells. Following a wash to remove any unbound avidin-enzyme reagent, a substrate solution was added to the wells, and color develops in proportion to the amount of CHIP bound in the initial step. The color development was stopped, and the intensity of the color was measured. The measurement of each sample was tested twice. The value of 58.68 pg/ml that could differentiate pancreatic cancer from chronic pancreatitis by Yoden index was determined as a cutoff point to measure the higher or lower expression of CHIP in serum.

### Statistical Methods

The values were presented as the mean ± SD. The two-tailed Student's t-test was used for comparing the mean values between two groups. χ2 or Fisher's exact test was used to compare categorical characteristics across groups. Univariate models and multivariable logistic regression was used to assess the significance of CHIP in the prognosis or prediction of pancreatic cancer. The survival rates were calculated by the method of Kaplan-Meier. A value of P<0.05 was considered to be statistically significant. SPSS 13.0 (SPSS Inc, USA) was used for statistical analysis.
